# Peripheral Kynurenine Pathway Metabolites in Patients with Psoriasis

**DOI:** 10.3390/ijms26073139

**Published:** 2025-03-28

**Authors:** Anna Stepaniuk, Anna Baran, Justyna Magdalena Hermanowicz, Beata Sieklucka, Dariusz Pawlak, Iwona Flisiak

**Affiliations:** 1 Department of Dermatology and Venerology, Medical University of Bialystok, Zurawia 14, 15-540 Bialystok, Poland; stepaniukanna@gmail.com (A.S.); iwona.flisiak@umb.edu.pl (I.F.); 2 Department of Pharmacodynamics, Medical University of Bialystok, Mickiewicza 2C, 15-089 Bialystok, Poland; justyna.hermanowicz@umb.edu.pl (J.M.H.); dariusz.pawlak@umb.edu.pl (D.P.); 3 Department of Monitored Pharmacotherapy, Medical University of Bialystok, Mickiewicza 2C, 15-089 Bialystok, Poland; beata.sieklucka@umb.edu.pl

**Keywords:** psoriasis, kynurenine pathway, tryptophan, kynurenine, kynurenic acid, indoleamine-2,3-dioxygenase

## Abstract

Psoriasis is a systemic disease affecting 2–3% of the general population. Tryptophan (TRP) is an amino acid metabolized in the kynurenine pathway (KP). The aim of this study was to assess the kynurenine pathway’s metabolites in serum and urine of psoriatic patients and explore the possible interplay with the disease’s pathogenesis and its comorbidities. The study involved 60 patients with plaque psoriasis and 30 healthy volunteers matched for gender, age, and BMI. Serum and urine samples were taken from the participants and tested for TRP, indoleamine 2,3-dioxygenase (IDO), 2,3-tryptophan dioxygenase (TDO), kynurenine (KYN), kynurenic acid (KYNA), quinolinic acid (QUIN), and numerous laboratory parameters. Correlations between the metabolites’ levels and clinical, laboratory parameters and depression occurrence were statistically evaluated. Concentrations of tryptophan, kynurenic acid, and quinolinic acid in serum and urine were significantly higher among patients with psoriasis (*p* < 0.05 and *p* < 0.001, *p* < 0.05 and *p* < 0.05 and *p* < 0.001 and *p* < 0.001, respectively). A significant stimulation of the kynurenine pathway in serum and urine of patients with psoriasis suggests its role in its pathogenesis and interplay between chronic inflammation or comorbidities. Further research is needed to discover whether the increase in KP metabolites is an indicator of inflammation or a compensatory mechanism in psoriasis.

## 1. Introduction

Psoriasis is a complex systemic disease commonly characterized by scaling, red plaques, which can also include nail and joint involvement, of still not fully understood pathogenesis related to numerous comorbidities such as hypertension, diabetes mellitus (DM), obesity, and depression [1]. Psoriasis has been recognized as systemic disease strongly related with numerous comorbidities, such as depression, hypertension, diabetes mellitus (DM), obesity, non-alcoholic fatty liver disease (NAFLD), myocardial infarction (MI), chronic kidney disease (CKD), and inflammatory bowel disease (IBD), among others [1,2,3]. The common coexistence of psoriasis and metabolic syndrome (MS), along with systemic inflammation, increased uric acid level, and the nephrotoxic effect of some of the psoriasis medications, leads to an increased risk of renal diseases, such as CKD and end-stage renal disease [3,4]. Cohort studies found that even mild psoriasis doubled the risk of death from kidney disease [3]. Psoriasis has a significant detrimental effect on patients’ quality of life (QoL) but also has psychosocial and economic burdens [5]. Depressive symptoms affect even up to 68% of psoriatics [5].

**Figure 1:**
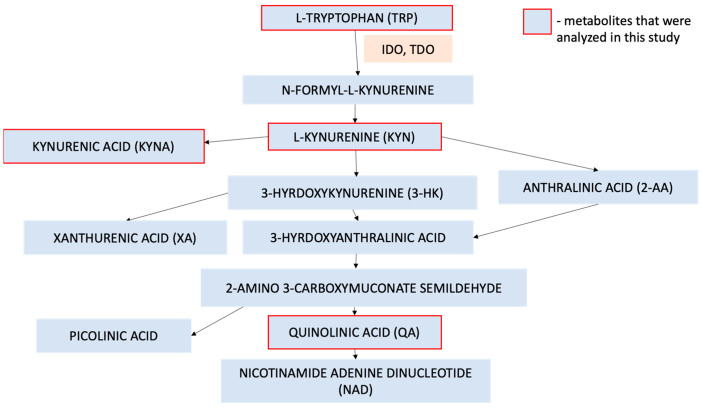
The kynurenine pathway.

Tryptophan (TRP) is an amino acid important in regulating inflammatory and stress responses, the gastrointestinal tract, and mental health [6]. TRP is catabolized in several metabolic pathways, one of them being kynurenine pathway (KP), where over 95% of tryptophan is metabolized (Figure 1) [6]. A link between another route of tryptophan catabolism via serotonin production has been proved [6]. Aberrations in the KP have been noted in numerous diseases that are also psoriasis comorbidities such as depression, atherosclerosis, obesity, and coronary artery disease (CAD) [6,7]. Higher quantities of KP metabolites, such as kynurenic acid, anthranilic acid and 3-hydroxykynurenine, were linked to an increased risk of acute MI [8]. The overactivity of two key enzymes—IDO and TDO—was observed among patients with depression [7]. Therefore, Correia et al. suggested that inhibitors of these enzymes could potentially be used in treating depression [7]. Furthermore, some studies reported that KP metabolites such as quinolinic acid can exhibit neurotoxic activity [6]. Mallmann et al. observed a higher kynurenine to tryptophan (KYN/TRP) ratio among patients with obesity and metabolic syndrome and both were significantly more frequent in patients with psoriasis [9,10]. Other studies found that the KYN/TRP ratio can forecast the risk of acute coronary events, such as MI, which occur more commonly among psoriatics [1,8]. That same ratio, along with kynurenine itself, was found to be a good marker for the development of CKD [11]. Elevated levels of kynurenic acid were also observed in acute kidney injury (AKI) and have been linked to a higher AKI stage and longer duration of the disease [12,13].

Studies on kynurenine pathway in psoriasis are very limited but demonstrate upregulation of KP in psoriasis patients and an increased activity of two enzymes—indoleamine 2,3-dioxygenase (IDO) and L-kynureninase (KYNU)—in psoriatic lesions [14]. Furthermore, we noted that available, but very limited, data on the incidence of KP upregulation in psoriasis do not include renal involvement; as kidney diseases are strongly connected to both KP and psoriasis, we decided to include renal parameters in our study. The same observation we made for depression; this disease commonly coexists with psoriasis and abbreviations in KP were noted but no studies further exploring this topic can be found. Therefore, we decided to evaluate KP metabolites levels and enzyme activities among patients with psoriasis and healthy volunteers and to assess those molecules in relation to depression incidence.

## 2. Results

Basic characteristics of the patients enrolled in the study and the control group are displayed in Table 1. The specific numerical values of the studied enzymes and metabolites can be found in the Supplementary File.

### 2.1. Tryptophan

#### 2.1.1. Serum Tryptophan

**Figure 2:**
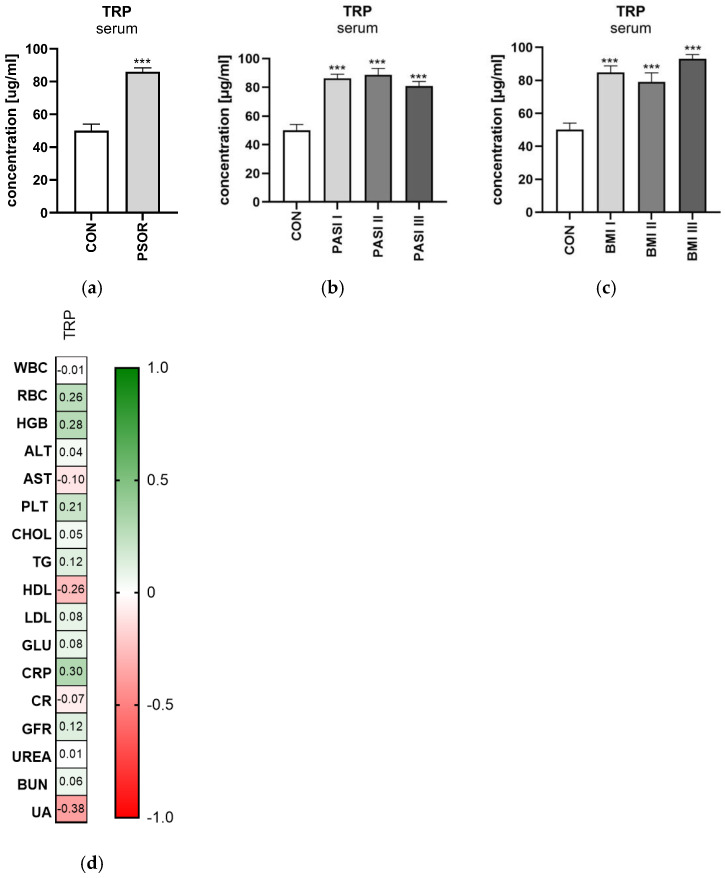
Serum tryptophan concentration in patients in the entire study group (**a**), depending on PASI (**b**), and depending on BMI (**c**), comparing to controls. (**d**) Correlation matrix between tryptophan concentration and basic laboratory parameters. Data are presented as mean ± SD (**a**–**c**) (*n* = 30 (CON); 60 (PSOR)). *** *p* < 0.001 for the comparison between controls (CON) and the PSOR group or appropriate PASI/BMI group by the Student *t*-test (**a**) or one-way ANOVA with Tukey’s post-hoc test (**b**,**c**). Matrix correlations were assessed using Spearman’s rank correlation analysis (**d**); the magnitude of the correlation is displayed in terms of color from negative (red) to positive correlation (green). Abbreviations: CON—controls; PSOR—psoriasis; TRP—tryptophan; PASI—Psoriasis Area and Severity index; BMI—body mass index; WBC—white blood cells; RBC—red blood cells; HGB—hemoglobin; ALT—alanine transaminase; AST—asparagine transaminase; PLT—platelets; CHOL—total cholesterol; TG—triglyceride; HDL—high-density lipoprotein; LDL—low-density lipoprotein; GLU—fasting glucose; CRP—C-reactive protein; CR—creatinine; GFR—glomerular filtration rate; BUN—blood urea nitrogen; and UA—uric acid.

Serum tryptophan concentration was significantly higher in the study group than in the healthy controls (*p* < 0.001) (Figure 2a). Serum tryptophan concentration was significantly higher in patients with mild, moderate and severe psoriasis than in the controls (all *p* < 0.001) (Figure 2b). Serum tryptophan concentration was significantly higher among all BMI subgroups compared to the controls (all *p* < 0.001) (Figure 2c). Serum TRP level was significantly higher among patients with psoriasis, regardless of the duration of the disease, when compared to the healthy individuals (mean 50.02; 85.95; 86.00 respectively, SD 21.90; 21.40; 15.94 respectively). Significant positive correlations between TRP and RBC, HGB, CRP were noted (R = 0.26, *p* = 0.05; R = 0.28, *p* = 0.03; and R = 0.30, *p* = 0.03, respectively) (Figure 2d).

#### 2.1.2. Urinary Tryptophan/Creatinine Ratio Concentration

**Figure 3:**
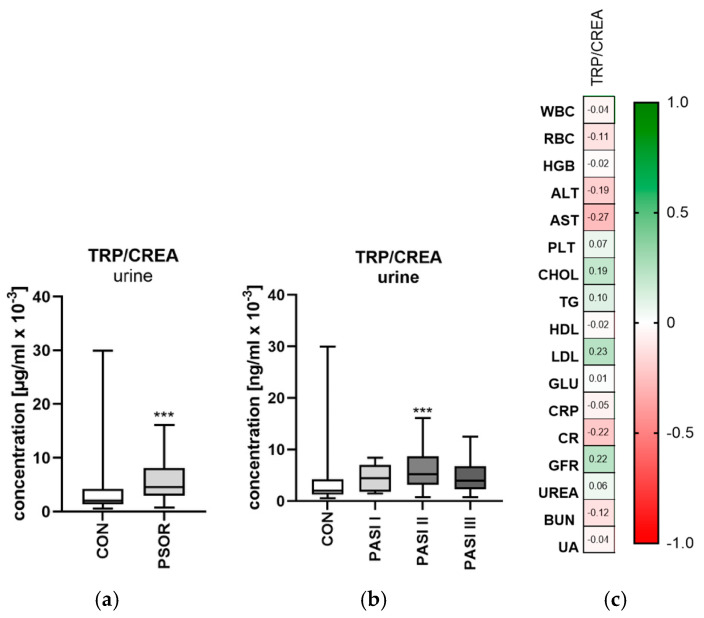
Urinary tryptophan/creatinine ratio concentration in patients in the entire study group (**a**) and depending on PASI (**b**) compared to the controls. (**c**) Correlation matrix between urinary tryptophan/creatinine concentration and basic laboratory parameters. Data are presented as median (full range) (**a**,**b**) (*n* = 30 (CON); 60 (PSOR)). *** *p* < 0.001 for the comparison between the controls (CON) and PSOR group or appropriate PASI group by the Mann–Whitney U test (**a**) or Kruskal–Wallis test with Dunn’s post-hoc test (**b**). Matrix correlations were assessed using Spearman’s rank correlation analysis (**c**); the magnitude of the correlation is displayed in terms of color from negative (red) to positive correlation (green). Abbreviations: CON—controls; PSOR—psoriasis; TRP—tryptophan; CREA—creatinine; PASI—Psoriasis Area and Severity index; WBC—white blood cells; RBC—red blood cells; HGB—hemoglobin; ALT—alanine transaminase; AST—asparagine transaminase; PLT—platelets; CHOL—total cholesterol; TG—triglyceride; HDL—high-density lipoprotein; LDL—low-density lipoprotein; GLU—fasting glucose; CRP—C-reactive protein; CR—creatinine; GFR—glomerular filtration rate; BUN—blood urea nitrogen; and UA—uric acid.

Urinary tryptophan/creatinine ratio concentration was significantly higher in the study group than in the healthy controls (*p* < 0.001) (Figure 3a). The ratio was significantly higher in patients with moderate psoriasis than in the controls (*p* < 0.001) (Figure 3b). Urinary tryptophan/creatinine ratio concentration was significantly higher in female patients compared to the healthy ones (*p* < 0.05). The ratio was significantly higher among all BMI-subgroups when compared to the controls (*p* < 0.01, *p* < 0.05, *p* < 0.05, respectively). Urinary TRP/CREA ratio was significantly higher among patients than among the control group when study group was divided by duration of the disease (both *p* < 0.01). A negative correlation of the ratio with AST activity was noted (R = −0.27, *p* = 0.04) (Figure 3c).

### 2.2. Indoleamine 2,3-Dioxygenase

#### 2.2.1. Serum Indoleamine 2,3-Dioxygenase

**Figure 4:**
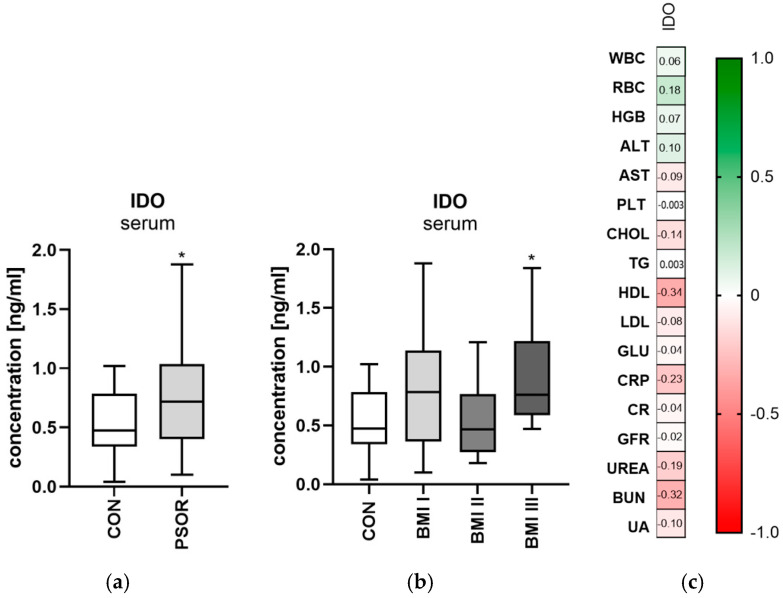
Serum indoleamine 2,3-dioxygenase concentration in all patients (**a**) and depending on BMI (**b**) in comparison to controls. (**c**) Correlation matrix between indoleamine 2,3-dioxygenase concentration and basic laboratory parameters. Data are presented as median (full range) (**a**,**b**) (*n* = 30 (CON); 60 (PSOR)). * *p* < 0.05 for the comparison between the controls (CON) and PSOR group or appropriate BMI group by the Mann–Whitney U test (**a**) or Kruskal–Wallis test with Dunn’s post-hoc test (**b**). Matrix correlations were assessed using Spearman’s rank correlation analysis (**c**); the magnitude of the correlation is displayed in terms of color from negative (red) to positive correlation (green). Abbreviations: CON—controls; PSOR—psoriasis; IDO—indoleamine 2,3-dioxygenase concentration; BMI—body mass index; WBC—white blood cells; RBC—red blood cells; HGB—hemoglobin; ALT—alanine transaminase; AST—asparagine transaminase; PLT—platelets; CHOL—total cholesterol; TG—triglyceride; HDL—high-density lipoprotein; LDL—low-density lipoprotein; GLU—fasting glucose; CRP—C-reactive protein; CR—creatinine; GFR—glomerular filtration rate; BUN—blood urea nitrogen; and UA—uric acid.

Serum indoleamine 2,3-dioxygenase concentration was significantly higher in the study group than in the healthy controls (*p* < 0.05) (Figure 4a). Serum indoleamine 2,3-dioxygenase concentration was significantly higher in female patients with psoriasis compared to the healthy volunteers (*p* < 0.01). Serum IDO was significantly higher among patients with a BMI > 30 compared to the controls (*p* < 0.05) (Figure 4b). A statistically significant negative correlation between IDO and HDL concentration was noted (R = −0.34, *p* = 0.03) (Figure 4c).

#### 2.2.2. Urinary Indoleamine 2,3-Dioxygenase/Creatinine Ratio Concentration

**Figure 5:**
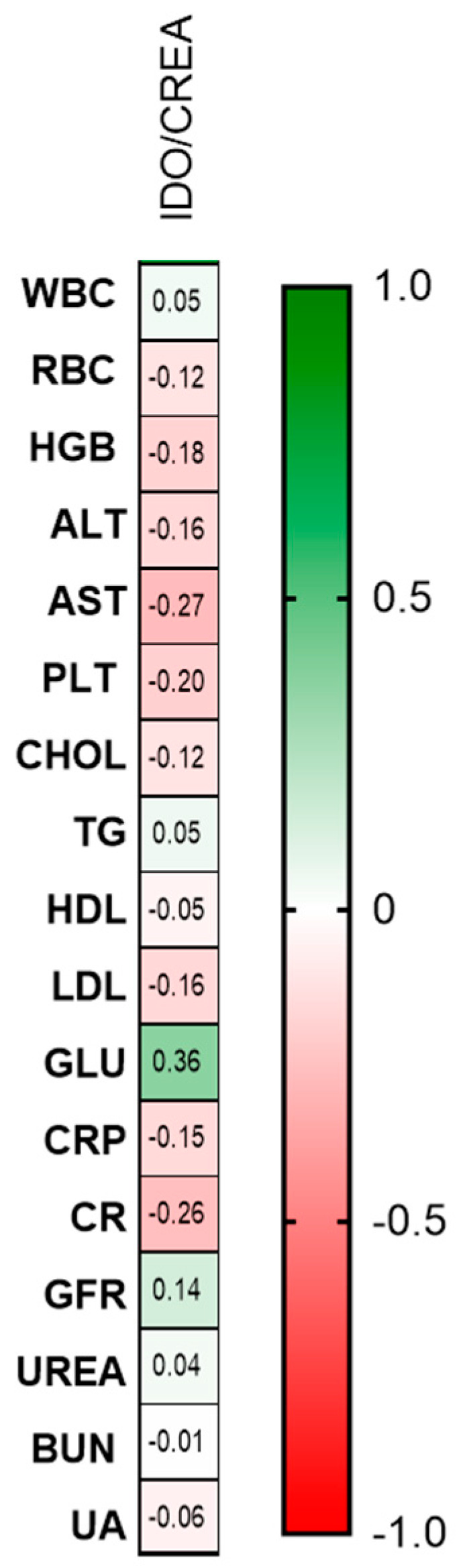
Correlation matrix between urinary indoleamine 2,3-dioxygenase/creatinine ratio concentration and basic laboratory parameters. Matrix correlations were assessed using Spearman’s rank correlation analysis; magnitude of the correlation is displayed in terms of color from negative (red) to positive correlation (green). Abbreviations: IDO—indoleamine 2,3-dioxygenase concentration; WBC—white blood cells; RBC—red blood cells; HGB—hemoglobin; ALT—alanine transaminase; AST—asparagine transaminase; PLT—platelets; CHOL—total cholesterol; TG—triglyceride; HDL—high-density lipoprotein; LDL—low-density lipoprotein; GLU—fasting glucose; CRP—C-reactive protein; CR—creatinine; GFR—glomerular filtration rate; BUN—blood urea nitrogen; and UA—uric acid.

Statistically significant negative correlations between AST activity (R = −0.27, *p* = 0.04) and CRP (R = −0.26, *p* = 0.05), and positive correlations between GLU (R = 0.36, *p* = 0.01) and indoleamine 2,3-dioxygenase/creatinine ratio concentration were noted (Figure 5).

### 2.3. Tryptophan 2,3-Dioxygenase

#### Serum Tryptophan 2,3-Dioxygenase

**Figure 6:**
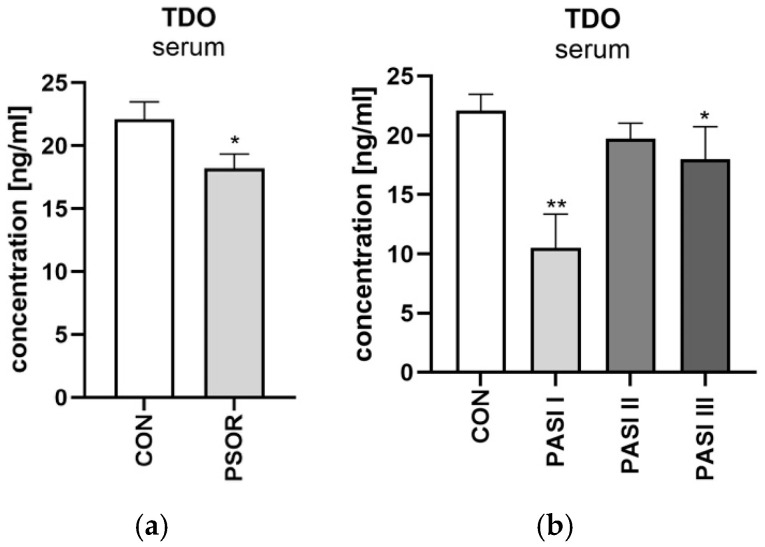
Serum tryptophan 2,3-dioxygenase activity in all patients (**a**) and depending on PASI (**b**) in comparison to controls. Data are presented as mean ± SD (**a**,**b**) (*n* = 30 (CON); 60 (PSOR)). * *p* < 0.05 and ** *p* < 0.01 for the comparison between the controls (CON) and PSOR group or appropriate PASI group by the Student *t*-test (**a**) or one-way ANOVA with Tukey’s post-hoc test (**b**). Abbreviations: CON—controls; PSOR—psoriasis; TDO—tryptophan 2,3-dioxygenase; and PASI—Psoriasis Area and Severity index.

Serum tryptophan 2,3-dioxygenase activity was significantly lower in the study group than in the healthy controls (*p* < 0.05) (Figure 6a). Serum tryptophan 2,3-dioxygenase concentration was significantly lower in patients with mild psoriasis and significantly higher in severe psoriasis than in the controls (*p* < 0.05; *p* < 0.01, respectively) (Figure 6b). Serum TDO activity was significantly lower among patients with long lasting psoriasis when compared to the healthy individuals (*p* < 0.5).

### 2.4. Kynurenine

#### 2.4.1. Serum Kynurenine

**Figure 7:**
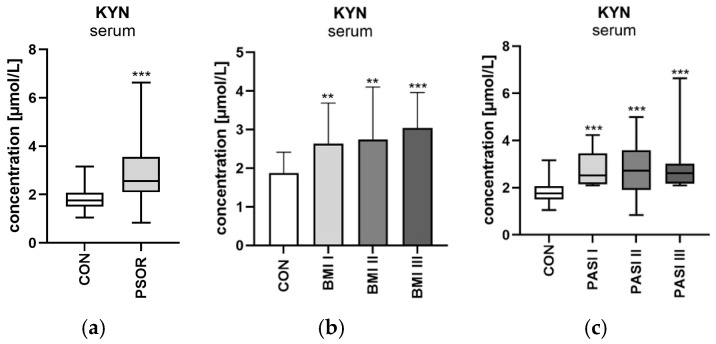
Serum kynurenine concentration in patients and controls (**a**). Division of serum kynurenine concentration in patients depending on BMI (**b**), and PASI (**c**) compared to controls. Data are presented as median (full range) (**a**,**c**) or mean ± SD (**b**) (*n* = 30 (CON); 60 (PSOR)). ** *p* < 0.01 and *** *p* < 0.001 for the comparison between the controls (CON) and PSOR group or appropriate PASI/BMI group by the Mann–Whitney U test (**a**), one-way ANOVA with Tukey’s post-hoc test (**b**), or the Kruskal–Wallis test with Dunn’s post-hoc test (**c**). Abbreviations: CON—controls; PSOR—psoriasis; KYN—kynurenine; PASI—Psoriasis Area and Severity index; and BMI—body mass index.

Serum kynurenine concentration was significantly higher in the study group than in the healthy controls (*p* < 0.001) (Figure 7a). KYN concentration was significantly higher among patients divided by BMI compared to the control group (*p* < 0.01, *p* < 0.01, *p* < 0.001 respectively) (Figure 7b). Serum kynurenine concentration was significantly higher in males compared to the healthy volunteers (*p* < 0.001). KYN was significantly higher in patients with mild, moderate, and severe psoriasis than in the controls (all *p* < 0.001) (Figure 7c). No statistically significant differences between serum kynurenine and laboratory parameters were noted.

#### 2.4.2. Urinary Kynurenine/Creatinine Ratio Concentration

**Figure 8:**
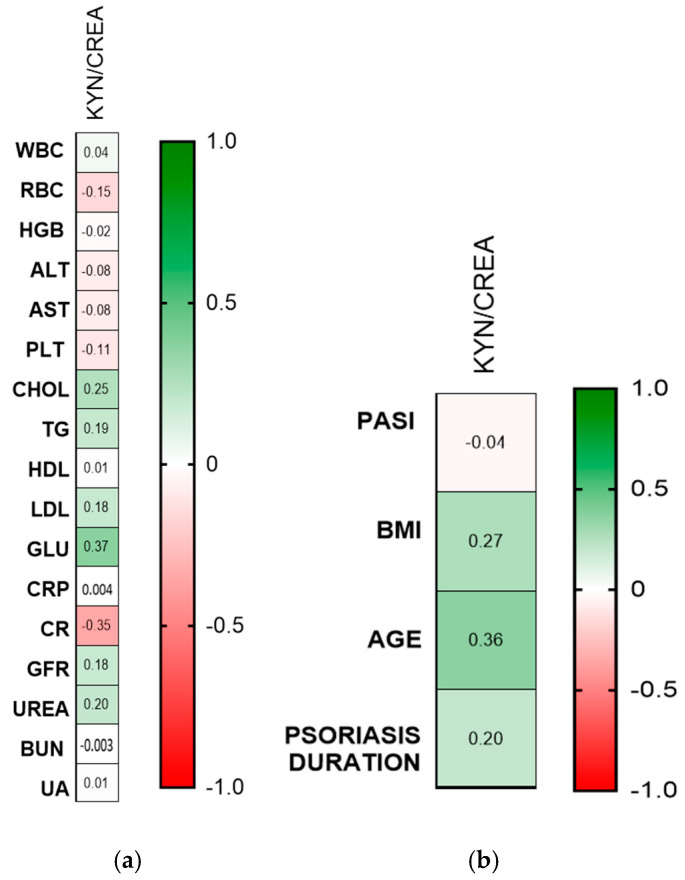
(**a**) Correlation matrix between urinary kynurenine/creatinine concentration and basic laboratory parameters. (**b**) Correlation matrix between kynurenine/creatinine ratio and PASI, BMI, age, and duration of the disease. Matrix correlations were assessed using Spearman’s rank correlation analysis; the magnitude of the correlation is displayed in terms of color from negative (red) to positive correlation (green). Abbreviations: KYN—kynurenine; CREA—creatinine; WBC—white blood cells; PASI—Psoriasis Area and Severity index; RBC—red blood cells; HGB—hemoglobin; ALT—alanine transaminase; AST—asparagine transaminase; PLT—platelets; CHOL—total cholesterol; TG—triglyceride; HDL—high-density lipoprotein; LDL—low-density lipoprotein; GLU—fasting glucose; CRP—C-reactive protein; CR—creatinine; GFR—glomerular filtration rate; BUN—blood urea nitrogen; and UA—uric acid.

Statistically significant positive correlations between kynurenine/creatinine ratio concentration and GLU, BMI, and age were noted (R = 0.27, *p* = 0.05; R = 0.36, *p* = 0.01; and R = 0.36, *p* = 0.01, respectively) (Figure 8a,b).

### 2.5. Kynurenic Acid

#### 2.5.1. Serum Kynurenic Acid

**Figure 9:**
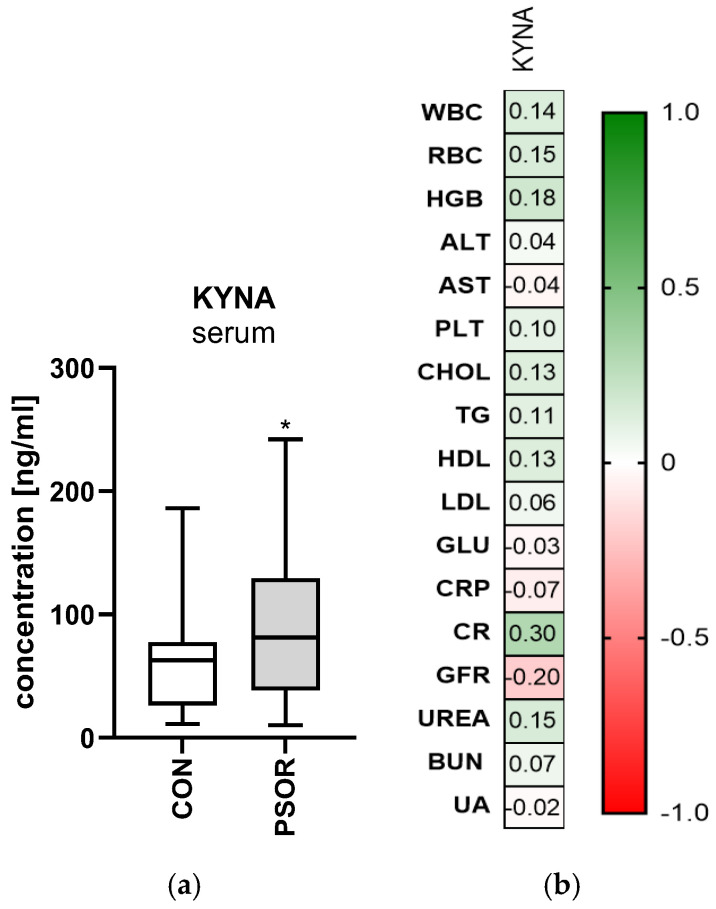
(**a**) Serum kynurenic acid concentration in patients and controls. (**b**) Correlations between serum kynurenic acid concentration and basic laboratory parameters. Data are presented as median (full range) (**a**) (*n* = 30 (CON); 60 (PSOR)). * *p* < 0.05 for the comparison between the controls (CON) and PSOR group by the Mann–Whitney U test (**a**). Matrix correlations were assessed using Spearman’s rank correlation analysis (**b**); the magnitude of the correlation is displayed in terms of color from negative (red) to positive correlation (green). Abbreviations: CON—controls; PSOR—psoriasis; KYNA—kynurenic acid; WBC—white blood cells; RBC—red blood cells; HGB—hemoglobin; ALT—alanine transaminase; AST—asparagine transaminase; PLT—platelets; CHOL—total cholesterol; TG—triglyceride; HDL—high-density lipoprotein; LDL—low-density lipoprotein; GLU—fasting glucose; CRP—C-reactive protein; CR—creatinine; GFR—glomerular filtration rate; BUN—blood urea nitrogen; and UA—uric acid.

Serum kynurenic acid concentration was significantly higher in the study group than in the healthy controls (*p* < 0.05) (Figure 9a). A statistically significant positive correlation between concentrations of kynurenic acid and CRP was noted (R = 0.30; *p* < 0.05) (Figure 9b).

#### 2.5.2. Urinary Kynurenic Acid/Creatinine Ratio Concentration

**Figure 10:**
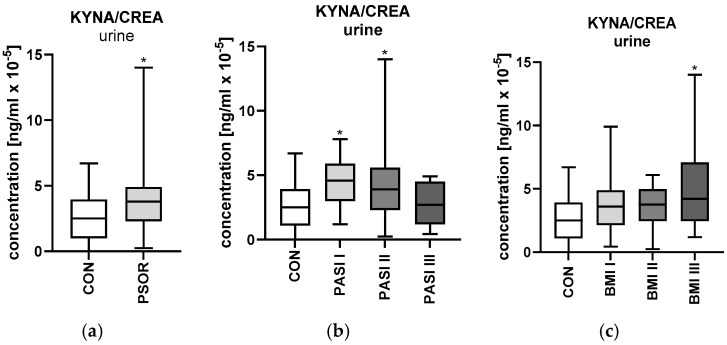
Urinary kynurenic acid/creatinine ratio concentration in all patients (**a**), depending on PASI (**b**), and depending on BMI (**c**), compared to the controls. Data are presented as median (full range) (**a**–**c**) (*n* = 30 (CON); 60 (PSOR)). * *p* < 0.05 for the comparison between the controls (CON) and PSOR group or appropriate PASI/BMI group by the Mann–Whitney U test (**a**) or the Kruskal–Wallis test with Dunn’s post-hoc test (**b**,**c**). Abbreviations: CON—controls; PSOR—psoriasis; KYNA—kynurenic acid; CREA—creatinine; and PASI—Psoriasis Area and Severity index.

Urinary kynurenic acid/creatinine ratio concentration was significantly higher in the study group than in the healthy controls (*p* < 0.05) (Figure 10a). The ratio was significantly higher among patients with mild and moderate psoriasis than in the controls (*p* < 0.05) (Figure 10b). Urinary KYNA/CREA concentration was significantly higher among females compared to the healthy controls (*p* < 0.05). The ratio was significantly the highest among obese patients compared to the control group and other BMI subgroups (*p* < 0.05) (Figure 10c). Urinary kynurenic acid/creatinine concentration was significantly higher in persons with shorter lasting psoriasis than in the controls (*p* < 0.05)

### 2.6. Quinolinic Acid

#### 2.6.1. Serum Quinolinic Acid

**Figure 11:**
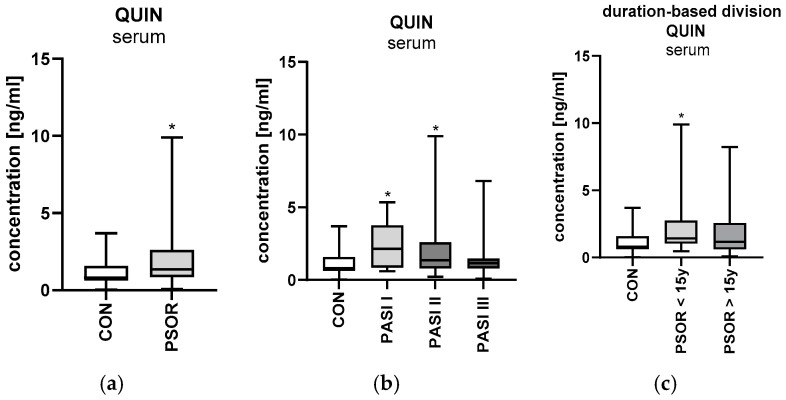
Serum quinolinic acid concentrations in all patients (**a**), depending on PASI (**b**) and depending on disease duration (**c**), compared to controls. Data are presented as median (full range) (**a**–**c**) (*n* = 30 (CON); 60 (PSOR)). * *p* < 0.05 for the comparison between the controls (CON) and PSOR group or appropriate PASI/PSOR </> 15 y group by the Mann–Whitney U test (**a**) or the Kruskal–Wallis test with Dunn’s post-hoc test (**b**,**c**). Abbreviations: CON—controls; PSOR—psoriasis; QUIN—quinolinic acid; and PASI—Psoriasis Area and Severity index.

Serum quinolinic acid concentration was significantly higher in the study group than in the healthy controls (*p* < 0.05) (Figure 11a). Serum quinolinic acid concentration was significantly higher in patients with mild and moderate psoriasis than in the controls (both *p* < 0.05) (Figure 11b). Serum QA levels were significantly higher in males compared to the healthy volunteers (*p* < 0.05). Serum quinolinic acid concentration was significantly higher among patients with a history of psoriasis for 15 years compared to the control group (*p* < 0.05) (Figure 11c).

#### 2.6.2. Urinary Quinolinic Acid/Creatinine Ratio Concentration

**Figure 12:**
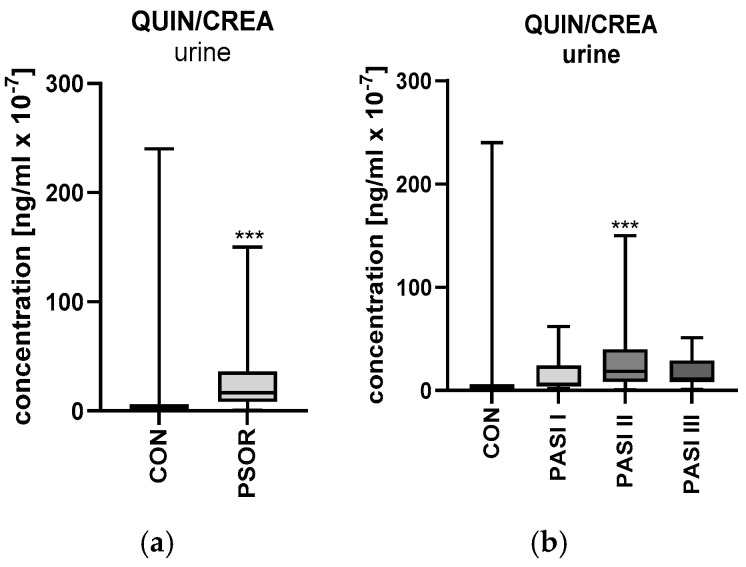

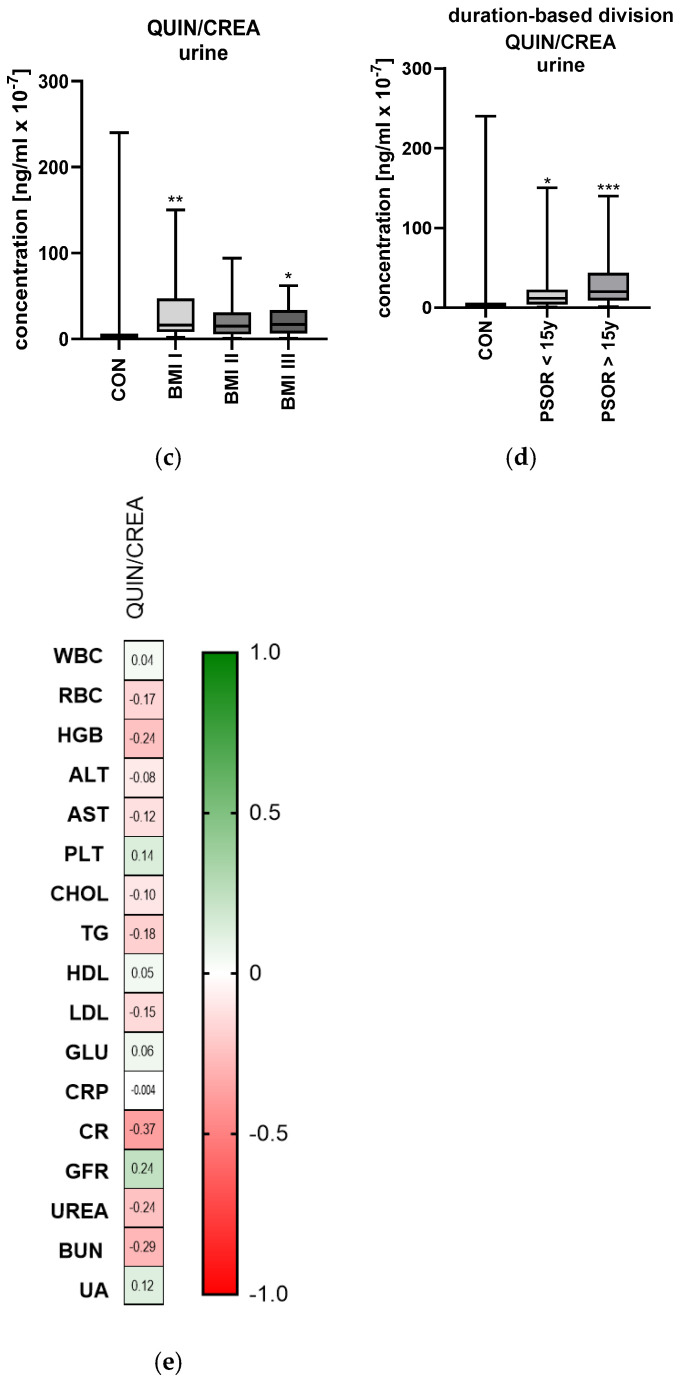
Urinary quinolinic acid/creatinine ratio concentration in all patients (**a**), depending on PASI (**b**), BMI (**c**), or duration of the disease (**d**) compared to the controls. (**e**) Correlation matrix between urinary quinolinic acid/creatinine concentration and basic laboratory parameters. Data are presented as median (full range) (**a**–**d**) (*n* = 30 (CON); 60 (PSOR)). * *p* < 0.05; ** *p* < 0.01; and *** *p* < 0.001 for the comparison between the controls (CON) and PSOR group or appropriate PASI/BMI/PSOR </> 15 y group by the Mann–Whitney U test (**a**) or the Kruskal–Wallis test with Dunn’s post-hoc test (**b**–**d**). Matrix correlations were assessed using Spearman’s rank correlation analysis (**e**); the magnitude of the correlation is displayed in terms of color from negative (red) to positive correlation (green). Abbreviations: CON—controls; QUIN—quinolinic acid; PSOR—psoriasis; CREA—creatinine; WBC—white blood cells; RBC—red blood cells; HGB—hemoglobin; ALT—alanine transaminase; AST—asparagine transaminase; PLT—platelets; CHOL—total cholesterol; TG—triglyceride; HDL—high-density lipoprotein; LDL—low-density lipoprotein; GLU—fasting glucose; CRP—C-reactive protein; CR—creatinine; GFR—glomerular filtration rate; BUN—blood urea nitrogen; and UA—uric acid.

Urinary quinolinic acid/creatinine ratio concentration was significantly higher in the study group than in the healthy controls (*p* < 0.001) (Figure 12a). The ratio was significantly higher in patients with moderate psoriasis than in the controls (*p* < 0.001) (Figure 12b). Urinary quinolinic acid/creatinine ratio concentration was significantly higher among patients with a BMI between 20 and 25 and a BMI higher than 30 when compared to the control group (*p* < 0.01 and *p* < 0.05, respectively) (Figure 12c). The ratio was significantly higher among patients with psoriasis, regardless of the duration of the disease, when compared to the healthy individuals (*p* < 0.05 and *p* < 0.001, respectively) (Figure 12d). A statistically significant negative correlation between CRP and QUIN/CREA concentration was noted (Figure 12e).

### 2.7. Beck’s Depression Inventory

**Figure 13:**
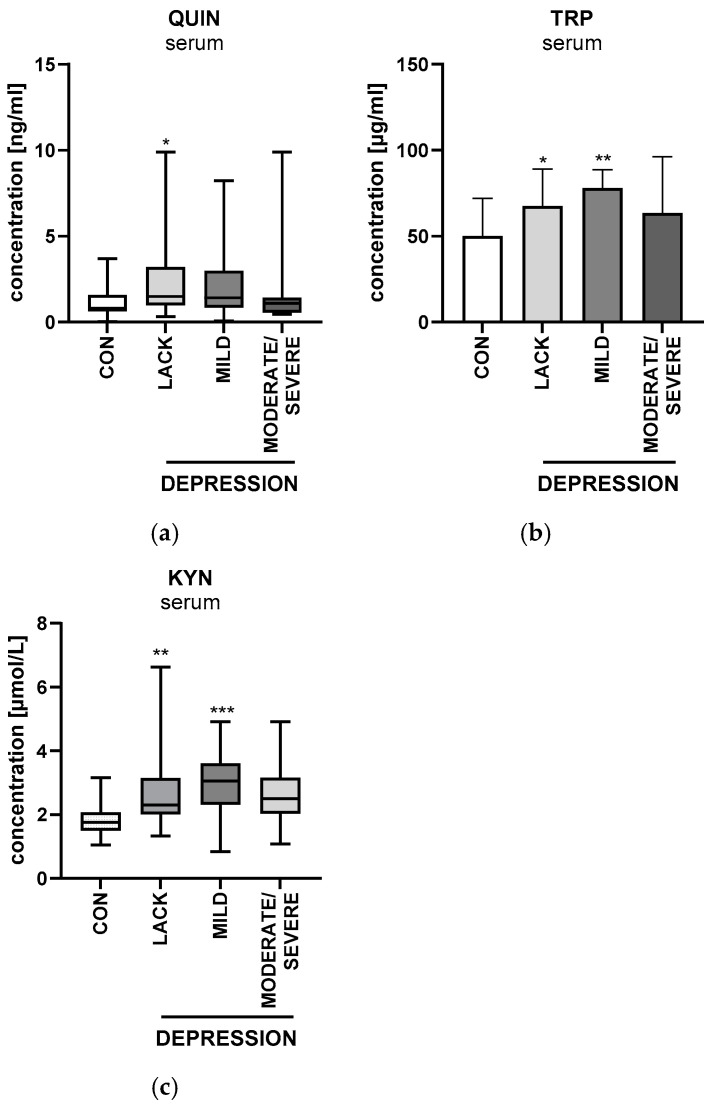
(**a**) Serum quinolinic acid concentration among patients with mild depression, moderate/severe depression, and without depression compared to the controls. (**b**) Serum tryptophan concentration among patients with mild depression, moderate/severe depression, and without depression compared to the controls. (**c**) Serum kynurenine concentration among patients with mild depression, moderate/severe depression, and without depression compared to the controls. Data are presented as median (full range) (**a**,**c**) and median ± SD (**b**) (*n* = 30 (CON); 60 (PSOR)). * *p* < 0.05; ** *p* < 0.01; and *** *p* < 0.001 for the comparison between the controls (CON) and PSOR group with mild depression, moderate/severe depression, or without depression by the Kruskal–Wallis test with Dunn’s post-hoc test (**a**,**c**) and one-way ANOVA with Tukey’s post-hoc test (**b**). Abbreviations: CON—controls; QUIN—quinolinic acid; TRP—tryptophan; and KYN—kynurenine.

Among the study group, 23 (38%) patients suffered from mild depression, 7 (12%) from moderate/severe depression, and 30 (50%) patients were without depression. Serum quinolinic acid concentration was significantly higher among psoriatic patients without depression than in the control group (*p* < 0.05) (Figure 13a). Serum tryptophan concentration was significantly higher among psoriatics without depression and the ones with a mild form compared to the controls (*p* < 0.05 and *p* < 0.01, respectively) (Figure 13b). Serum kynurenine concentration was significantly higher among patients without depression and with mild depression compared to the control group (*p* < 0.01 and *p* < 0.001, respectively) (Figure 13c).

## 3. Discussion

The kynurenine pathway is a key immune regulator and increased tryptophan metabolism has been linked to systemic inflammation [6]. In our study a similar correlation among patients with psoriasis, as to the one of inflammatory diseases, was observed. We noted significantly higher activities of enzymes and concentrations of metabolites of the KP than in the healthy persons, confirming its uncertain role in psoriasis.

A significant correlation between TRP and CRP suggests its potential role as an inflammatory marker. We noted significantly higher tryptophan concentrations in both serum and urine among the study group compared to the healthy controls. TRP, which cannot be synthetized by humans, is provided by dietary consumption and is later metabolized by liver tissue [15]. It is believed that this substance regulates biological functions such as sleep and appetite, and alterations in TRP metabolism can lead to diseases such as depression [16]. Furthermore, increased TRP levels have been observed in inflammatory diseases related to psoriasis, such as inflammatory bowel disease [16]. A significant correlation between TRP and CRP suggests its potential role as an inflammatory marker. On the other hand, presumably, tryptophan’s role is more sophisticated in psoriasis and takes part in some compensatory mechanisms to diminish the chronic inflammation. In our study, kynurenine was significantly higher among psoriatics than in the healthy controls. Similarly, IDO and TDO were overactivated, resulting in increased KYN levels, which is in line with others [14,17]. Harden et al. observed increased IDO and KYNU activity in lesional skin compared to non-lesional skin and to skin biopsies from the control group [14]. Fujii et al. evaluated skin samples from patients with psoriasis, healthy volunteers, and mice for the presence of IDO1 and its isoform, IDO2, which is present in monocytes and dendritic cells [18]. Samples for both psoriatics and healthy volunteers were negative for IDO1 [18]. However, the expression of IDO2 in the epidermis of lesional and non-lesional skin of the patients was noted [18]. Skin samples of healthy individuals were mainly negative but some of them expressed weak signal [18].

Fujii et al. also evaluated KP activity in mice, where they noted a significantly higher IDO2 activity in the epidermis after psoriasis induction [18]. Most relevant was that a psoriasis-like skin inflammation was significantly worse in the IDO2 knockout mice [18]. The authors pointed to an IDO2 immunosuppressive effect by suppressing IL-17 expression [18]. Considering IL-17 inhibitors as effective antipsoriatic drugs, IDO2 and IL-17 interplay could be a newer therapeutic pathway [18]. Different outcomes were obtained by Choudhary et al. who analyzed mice with psoriasis-like lesions without the IDO1 gene [19]. PASI scores, epidermal thickness, and ear edema in comparison to wild-type mice were not of statistical significance [19]. Therefore, they emphasized that lack of influence of the KP on inflammatory processes in psoriasis should be considered, and potential compensatory changes in activity of other metabolic routes or IDO2 activity should be examined [19]. Krupa et al. highlighted that IDO2 activity is approximately 500–1000 times lower than IDO1, but it can express proinflammatory activity and can lead to autoreactivity of B cells or even aggravate the symptoms of autoimmune diseases such as arthritis [15].

One of the main goals of this study was to assess the prevalence of depression and concentrations of KP enzymes and metabolites in relation to this disease. The prevalence of depression among our patients was 50%, which is within the range found in the literature (between 24 and 90%), while in the general population, individuals with depression total 6–8% [5]. Higher plasma concentrations of the KYN/KYNA ratio and QA in patients with depression were documented [6,7]. In our study, contrary to the literature data, QA and KYN were significantly higher among patients without depression. Correia et al. noted the hyperactivity of IDO and TDO in depression and contributed these changes to the loss of astrocytes, which later causes the increased activity of the 3HK pathway [7]. This leads further to apoptosis-releasing astrocytes, among others, including QA, which has neurotoxic properties [7]. In our research, we also observed increased IDO activity but not TDO activity. These outcomes point to the possible role of KP metabolites in the interplay between psoriasis and depression. One of the possible links may be neurotoxicity of some metabolites of the KP. QA is believed to exhibit such effect by, among others, destabilizing the blood–brain barrier, affecting autophagy, and creating reactive oxygen molecules, whereas 3HK is known to generate free radicles [6].

Alterations in the kynurenine pathway have also been observed in other diseases that are considered psoriasis comorbidities. Increased intestinal IDO activity, which as a result upregulates the KP, has been observed among patients with obesity [20]. It is believed that this correlation happens due to chronic inflammation among this group [21]. In our study, urinary KYNA/CREA was the highest in obese psoriatics, which points to interesting role in the interplay with obesity that should be further explored. Elevated levels of KP metabolites have been observed in hypertension, atherosclerosis, and acute myocardial infarction, which all commonly coexist with psoriasis and have a common inflammatory and immunological basis [22]. Yang et al. suggested that those metabolites are ligands to the AhR receptor, which stimulates myocardial hypertrophy and hypervascularization, and therefore leads to development of cardiovascular diseases [22]. In our study, we also noted another correlation between the cardiovascular system, TRP, and KP-positive relations of TRP with morphotic parameters, which suggest tryptophan’s role in erythropoesis in psoriasis. Chronic kidney disease, affecting more often patients with psoriasis, is also linked with the KP. Pan et al. observed that IDO can predict the development of CKD with a sensitivity as high as 83.8% [23]. Psoriasis is also associated with NAFLD, where increased IDO activity and higher concentrations of metabolites of the KP were reported [24,25]. Teunis et al. noted that IDO is activated by molecules such as IL-6 and INF, which have proinflammatory properties [25]. Therefore, IDO is linked to inflammation, and as a result, to fibrosis [25]. The data strongly point to KP involvement in numerous autoimmune diseases, which also include psoriasis; however, it is still understudied.

There is still lack of knowledge of how to change KP activity. However, Tillmann et al. obtained some promising results from a study in an animal model with a methyl-deficient diet (MDD) [26]. Elevated plasma KA, KYN, and QA were observed among rats on a MDD [26]. After administration of a probiotic, the animals had a tendency to lower their kynurenic acid level [26]. There are studies from the 1960s where a diet low in tryptophan resulted in skin lesion clearance. Modulating tryptophan intake and KP metabolite levels could be a promising therapeutic strategy in psoriasis [27].

This study has certain limitations, such as not a diverse enough study group, with high male predominance and originating from one city and of one ethnicity. Serum and urine samples were collected once from each participant, and they were not advised to follow any specific diet before enrolling in the study. Therefore, fluctuations due to different dietary consumption of tryptophan cannot be ruled out. Regarding depression prevalence, it is worth noting that patients hospitalized in the department were included in the study and the prevalence of depression was assessed while they were in the department. Therefore, a detrimental effect of a hospital setting cannot be ruled out.

## 4. Materials and Methods

The study group consisted of 60 patients with exacerbation of plaque psoriasis. The control group of 30 healthy volunteers without any skin diseases was matched according to age, gender, and body mass index (BMI). Pregnancy, breastfeeding, or anti-psoriasis treatment in the four weeks before enrolling in the study and hypertension, chronic kidney or heart failure, liver disease, acute and chronic infection, other autoimmune diseases, and malignant tumors were the main exclusion criteria. The severity of the psoriatic lesions was assessed by the same investigator in all patients using the Psoriasis Area and Severity index (PASI) on admission to the department.

The study group was divided according to the PASI into three subgroups: mild (PASI 1), meaning scored under 10 points and noted in 9 patients; moderate (PASI 2), a PASI of 10–20 points, evaluated in 41 subjects; and severe psoriasis (PASI 3) was related to a PASI > 20 points, calculated in 10 persons. BMI was evaluated as weight/height 2 (kg/m^2^). The study group was further divided according to BMI: group 0 meant the control group; a BMI 1–10, normal weight (18.5–24.9), patients with psoriasis; BMI 2, indicated in 10 overweight psoriatics (BMI 25–29.9); BMI 3, obesity (BMI > 30). The study group was also divided in relation to depression severity using the Beck’s depression inventory.

Serum and urine samples were collected from the participants. Both serum and urine samples were taken from the participants in the morning upon waking up, before breakfast and after night rest. This time of the day was chosen as samples for assessing the KP and regular blood and urine tests were taken at the same time to avoid unnecessary injections, and particularly, the results of the standard blood and urine tests can dramatically differ when the samples are taken later in the day or after a meal. They were stored at −80 °C until final analysis. The concentrations of the tested TRP metabolites: TRP (Tryptophan ELISA kit, Cloud-Clone Corp., Katy, TX, USA), KYN (IDK Kynurenine ELISA kit, Immundiagnostik AG, Bensheim, Germany), KYNA (Kynurenic acid ELISA kit, Cloud-Clone Corp., Katy, TX, USA), QA (Quinolinic acid ELISA kit, Cloud-Clone Corp., Katy, TX, USA), TDO (Tryptophan-2,3-dioxygenase ELISA kit, Cloud-Clone Corp., Katy, TX, USA), IDO (Indoleamine-2,3-Dioxygense ELISA kit, Cloud-Clone Corp., Katy, TX, USA), and CREA (Urinary creatinine detection kit, Arbour assays, Ann Arbor, MI, USA) were determined using commercially available ELISA tests. All ELISA tests were done following manufacturer’s instructions. Briefly, for the assessment of KYN, the sample preparation included the addition of a derivatization reagent. Afterwards, the samples were incubated with monoclonal or polyclonal antibodies specific to appropriate metabolites in the wells of a microtiter. After incubation, the unbound conjugate was washed off. Next, avidin conjugated to horseradish peroxidase (HRP) or tetramethylobenzidine (TMB) was added to each microplate well and incubated. The amount of bound HRP or TMB conjugate was reverse proportional to the concentration of TRP, KYN, KYNA, and QA or directly proportional to the concentration of IDO, TDO, and CREA in the sample. We assessed the risk of depression among the study group using the Beck’s depression inventory. Quinolinic acid, kynurenine, indoleamine 2,3-dioxygenase, and L-kynureninase were assessed in serum and urine samples taken from study and control groups. Additional standard laboratory metrics were obtained from the psoriatics.

The normality of the distribution was assessed by the Shapiro–Wilk test. Normally distributed data were presented as means ± SD (standard deviations), whereas data lacking normal distributions were reported as medians (full range). In the statistical analysis, the Student *t*-test was used to compare normally distributed data, whereas the Mann–Whitney U test was used for the non-parametric data. A multiple-group comparison between parametric data was made by one-way analysis of variance (ANOVA), and significant differences between the groups were assessed using Tukey’s post-hoc test at *p*  <  0.05. The Kruskal–Wallis test with Dunn’s post-hoc test was used for nonparametric data at *p*  <  0.05. The correlations were analyzed using a Spearman’s rank correlation analysis. A *p*-value < 0.05 was considered statistically significant. Graphic design presentation of the results was prepared using GraphPad Prism 6 (GraphPad Software, La Jolla, CA, USA) or Statistica ver.10 computer software (StatSoft, Tulsa, OK, USA).

## 5. Conclusions

This study sheds a unique light on correlations between metabolites and enzymes of the kynurenine pathway in both blood and urine in relation to depression incidence, which remains an understudied topic in psoriasis. To our knowledge, this is the first study which analyzed KP metabolism in urine of patients with psoriasis and in relation to depression incidence or laboratory and clinical data. A significant overactivation of the KP demonstrated its undeniable role in psoriasis. The TRP metabolism pathway might be not only a marker of psoriasis and its inflammation but even a component in compensatory mechanisms, which should be explored. Modulating the effect of tryptophan or dietary supplementation could influence the psoriasis course. Our promising results suggest that it might be valuable to consider further clinical studies on a larger number of participants that involves the implementation of low tryptophan to assess its influence on both psoriasis activity and depression’s severity, which could later potentially lead to a new treatment option in those diseases.

## Figures and Tables

**Table 1:** Basic characteristics of patients and controls. Data are presented as mean ± SD or median (full range) depending on their distribution (*n* = 30 (controls); 60 (study group)). NS, non significant.

Parameter	Controls (*n* = 30)	Study Group (*n* = 60)
Sex (M/F)	21/9	43/17 NS
Age [years]	43 ± 2	47 ± 2 NS
Height [m]	1.7 ± 0.1	1.71 ± 0.1 NS
Weight [kg]	77.5 (67–90)	83 (57–120) NS
BMI ratio	25.9 ± 0.4	27.8 ± 0.6 NS

## Data Availability

Data is contained within the article and Supplementary Materials.

## Supplementary Materials

The following supporting information can be downloaded at: https://www.mdpi.com/article/10.3390/ijms26073139/s1.

## Abbreviations

WBC, white blood cells; RBC, red blood cells; HGB, hemoglobin; ALT, alanine transaminase; AST, asparagine transaminase; PLT, platelets; Chol, total cholesterol; TG, triglycerides; HDL, high-density lipoprotein; LDL, low-density lipoprotein; GLU, fasting glucose; CRP, C-reactive protein; CR, creatinine; GFR, glomerular filtration rate; BUN, blood urea nitrogen; and UA, uric acid */**/*** means a statistically significant difference compared to the control group with *p* < 0.05/0.01/0.001, respectively.
