# Supplementary material for: Peripheral Kynurenine Pathway Metabolites in Patients with Psoriasis
Source: Int J Mol Sci. 2025 Mar 28;26(7):3139. doi: 10.3390/ijms26073139 (PMC11988929; doi:10.3390/ijms26073139)
Supplement: Supplementary file 1 [file ijms-26-03139-s001.zip › ijms-3494967-supplementary.pdf]

1. Tryptophan
1.1 Serum tryptophan

	CON	PSOR
Mean	50,02	85,98
Std. Deviation	21,90	18,47
Std. Error of Mean	4,066	2,404

	CON	PASI I	PASI II	PASI III
Mean	50,02	86,33	88,60	80,85
Std. Deviation	21,90	11,48	23,90	12,66
Std. Error of Mean	4,066	2,785	4,599	3,268

	CON FEMALE	PSOR FEMALE	CON MALE	PSOR MALE
Mean	45,19	89,12	51,85	84,70
Std. Deviation	23,43	12,21	21,59	20,46
Std. Error of Mean	8,283	2,961	4,712	3,157

	CON	BMI I	BMI II	BMI III
Mean	50,02	84,77	79,05	93,06
Std. Deviation	21,90	17,64	23,23	11,72
Std. Error of Mean	4,066	3,945	5,475	2,558

	CON	<15 years	>15 years
Mean	50,02	85,95	86,00
Std. Deviation	21,90	21,40	15,94
Std. Error of Mean	4,066	4,119	2,817

	R VALUE	P VALUE
WBC	-0,00976137248834206	0,941506923575911
RBC	0,259784297957854	0,0469218145282322*
HGB	0,280952630900062	0,0311238327431873*
ALT	0,0363593990841511	0,784548298959699
AST	-0,103485730889212	0,435403257801912
PLT	0,210206351510775	0,110045208704069
CHOL	0,0479949118843993	0,748694643834171
TG	0,117789922299423	0,430385011291728
HDL	-0,261225995037822	0,0947211196597224
LDL	0,0829241179999126	0,615753379349924
GLU	0,0785896448760696	0,564792133707245
CRP	0,299244028367028	0,0295006986651757*
CRP	-0,0744499855257356	0,582047048358376
GFR	0,122117612759304	0,365499734875426
UREA	0,00588235294117647	0,98691165861886
BUN	0,0628375362078965	0,823986640527315
UA	-0,375853299436091	0,0847324510780932

	R VALUE	P VALUE
PASI	-0,174838921245912	0,197461200757377
BMI	0,256315843333079	0,0490488938132462*
AGE	-0,0975003748188291	0,462556486803472
PSORIASIS DURATION	0,034950730597048	0,792706361252159

1.2 Urinary tryptophan/creatinine ratio concentration

	CON	PSOR
Minimum	0,5415	0,7501
25% Percentile	1,313	2,962
Median	2,004	4,537
75% Percentile	4,206	8,095
Maximum	29,97	16,10
Range	29,42	15,35

	CON	PASI I	PASI II	PASI III
Minimum	0,5415	1,487	0,7501	0,7986
25% Percentile	1,313	1,825	3,175	2,329
Median	2,004	4,460	5,212	3,935
75% Percentile	4,206	7,048	8,717	6,748
Maximum	29,97	8,435	16,10	12,52
Range	29,42	6,948	15,35	11,72

	CON FEMALE	PSOR FEMALE	CON MALE	PSOR MALE
Minimum	0,9955	1,200	0,5415	0,2400
25% Percentile	1,072	3,250	1,301	2,175
Median	2,606	4,600	1,986	3,350
75% Percentile	4,788	6,450	3,979	4,800
Maximum	5,340	14,00	29,97	9,900
Range	4,344	12,80	29,42	9,660

	CON	BMI I	BMI II	BMI III
Minimum	0,5415	0,7986	0,9928	0,7501
25% Percentile	1,313	3,127	2,635	2,930
Median	2,004	5,610	5,048	4,323
75% Percentile	4,206	7,951	8,100	8,625
Maximum	29,97	16,10	13,06	13,77
Range	29,42	15,30	12,07	13,02

	CON	<15 years	>15 years
Minimum	0,5415	0,7501	0,7986
25% Percentile	1,200	3,170	2,875
Median	1,986	4,610	4,537
75% Percentile	4,155	7,993	8,625
Maximum	29,97	16,10	15,55
Range	29,42	15,35	14,75

	R VALUE	P VALUE
WBC	-0,03980535727282	0,764690157210214
RBC	-0,114225589156086	0,38899840100831
HGB	-0,0157595450156243	0,905696340520035
ALT	-0,19142068190401	0,146407357470171
AST	-0,269367270108685	0,0391047571212573*
PLT	0,0659086933616237	0,619921047259084
CHOL	0,194466130924379	0,190250569522703
TG	0,102581896003523	0,492623425942675
HDL	-0,0197136387559598	0,901382318426082
LDL	0,231357276715263	0,156445527427068
GLU	0,0142268460698194	0,917115421705698
CRP	-0,0514061083492267	0,714693368267188
CRP	-0,216702636440981	0,105419988094504
GFR	0,220155239775466	0,099852199704941
UREA	0,0558823529411765	0,839273736512514
BUN	-0,123879714238424	0,658169096115525
UA	-0,0411812146457842	0,852003256659726

	R VALUE	P VALUE
PASI	0,0795064185372418	0,560246340186707
BMI	-0,0758631774361729	0,567950677514356
AGE	-0,1375530025555	0,298848134726115
PSORIASIS DURATION	-0,0268784279654285	0,839857153094275

2. Indoleamine 2,3-dioxygenase

2.1 Serum indoleamine 2,3-dioxygenase

	CON	PSOR
Minimum	0,04000	0,1000
25% Percentile	0,3400	0,4000
Median	0,4750	0,7200
75% Percentile	0,7850	1,035
Maximum	1,020	1,880
Range	0,9800	1,780

	CON	PASI I	PASI II	PASI III
Mean	0,5440	0,7550	0,7558	0,8471
Std. Deviation	0,2792	0,4581	0,4345	0,4839
Std. Error of Mean	0,05097	0,1145	0,08522	0,1293

	CON FEMALE	PSOR FEMALE	CON MALE	PSOR MALE
Minimum	0,04000	0,3700	0,2200	0,1000
25% Percentile	0,2300	0,5400	0,3600	0,3400
Median	0,3600	0,7900	0,6300	0,6500
75% Percentile	0,6200	1,465	0,9100	1,010
Maximum	0,8000	1,880	1,020	1,840
Range	0,7600	1,510	0,8000	1,740

	CON	BMI I	BMI II	BMI III
Minimum	0,04000	0,1000	0,1800	0,4700
25% Percentile	0,3400	0,3625	0,2725	0,5900
Median	0,4750	0,7850	0,4650	0,7600
75% Percentile	0,7850	1,140	0,7675	1,220
Maximum	1,020	1,880	1,210	1,840
Range	0,9800	1,780	1,030	1,370

	CON	<15 years	>15 years
Minimum	0,04000	0,1000	0,1800
25% Percentile	0,3400	0,4150	0,4000
Median	0,4750	0,7550	0,6900
75% Percentile	0,7850	0,9675	1,160
Maximum	1,020	1,630	1,880
Range	0,9800	1,530	1,700

	R VALUE	P VALUE
WBC	0,0562912297268294	0,677480220140133
RBC	0,180463925638523	0,179159683564276
HGB	0,0659985798068345	0,625711646417243
ALT	0,104910572008614	0,43736145676999
AST	-0,0889243420360729	0,510669682607973
PLT	-0,00270699605055815	0,984055644813046
CHOL	-0,137238571425344	0,363088953362836
TG	0,00317724721438761	0,983280651551763
HDL	-0,335645302993544	0,0319220609915545*
LDL	-0,0846334922163775	0,613421245926022
GLU	-0,0370222071567126	0,790407234254902
CRP	-0,226295542904905	0,110310196455488
CRP	-0,0407898083107271	0,767474331770254
GFR	-0,0200068555292679	0,884725668022162
UREA	-0,193833069323353	0,503652578682341
BUN	-0,317241681086166	0,287700665461082
UA	-0,100265718939794	0,674052846474761

	R VALUE	P VALUE
PASI	-0,0970736823625534	0,484987454548943
BMI	0,153671621493564	0,253750825165808
AGE	-0,151830602778508	0,259551655302692
PSORIASIS DURATION	0,00111918538850917	0,99340758292566

2.2 Urinary indoleamine 2,3-dioxygenase /creatinine ratio concentration

	CON	PSOR
Minimum	0,06500	0,1200
25% Percentile	1,025	4,200
Median	6,350	11,00
75% Percentile	29,00	16,00
Maximum	120,0	72,00
Range	119,9	71,88

	CON	PASI I	PASI II	PASI III
Minimum	0,06500	3,000	0,1200	2,800
25% Percentile	1,025	4,200	2,546	4,200
Median	6,350	14,00	8,200	6,600
75% Percentile	29,00	16,00	16,00	11,00
Maximum	120,0	21,00	37,00	72,00
Range	119,9	18,00	36,88	69,20

	CON FEMALE	PSOR FEMALE	CON MALE	PSOR MALE
Minimum	4,300	0,2600	0,06500	0,1200
25% Percentile	5,350	7,200	0,3700	3,000
Median	9,200	14,00	4,800	8,200
75% Percentile	65,50	18,50	21,00	16,00
Maximum	97,00	50,00	120,0	72,00
Range	92,70	49,74	119,9	71,88

	CON	BMI I	BMI II	BMI III
Minimum	0,06500	0,1200	0,7200	0,2600
25% Percentile	1,025	2,575	5,100	5,400
Median	6,350	7,700	10,60	13,00
75% Percentile	29,00	16,00	20,00	16,00
Maximum	120,0	50,00	37,00	72,00
Range	119,9	49,88	36,28	71,74

	CON	<15 years	>15 years
Minimum	0,06500	0,2600	0,1200
25% Percentile	1,200	3,850	5,000
Median	6,100	9,600	11,00
75% Percentile	22,00	17,00	16,00
Maximum	120,0	50,00	72,00
Range	119,9	49,74	71,88

	R VALUE	P VALUE
WBC	0,0462915713396045	0,727726654708255
RBC	-0,116055262355106	0,38139596127259
HGB	-0,178853116252822	0,175302809927162
ALT	-0,156958616163156	0,235148005163589
AST	-0,269157583246815	0,0392634830506067*
PLT	-0,195462709151177	0,13791346580611
CHOL	-0,115353504421795	0,440048386447327
TG	0,0532623458308914	0,722163939047056
HDL	-0,047994153629613	0,762785510647472
LDL	-0,157331904928653	0,338796851064287
GLU	0,362170515238982	0,00608987667524474**
CRP	-0,153753096929304	0,271680732798813
CRP	-0,261384687313306	0,0495321819961398*
GFR	0,142871113891652	0,289050571657763
UREA	0,0382352941176471	0,891017946963001
BUN	-0,00718143270947388	0,982042114851639
UA	-0,0567699837133758	0,801865265262043

	R VALUE	P VALUE
PASI	-0,0827673806282969	0,544218149722735
BMI	0,152682980452776	0,248306995105837
AGE	0,100324819329619	0,449630046518462
PSORIASIS DURATION	0,00219555035505109	0,986832691867947

3. Tryptophan 2,3-dioxygenase

3.1 Serum tryptophan 2,3-dioxygenase

	CON	PSOR
Mean	22,10	18,18
Std. Deviation	7,316	8,894
Std. Error of Mean	1,359	1,158

	CON	PASI I	PASI II	PASI III
Mean	22,10	10,49	19,69	18,00
Std. Deviation	7,316	8,049	8,592	8,201
Std. Error of Mean	1,359	2,846	1,326	2,734

	CON FEMALE	PSOR FEMALE	CON MALE	PSOR MALE
Mean	19,33	16,29	23,35	18,95
Std. Deviation	4,062	6,283	8,165	9,717
Std. Error of Mean	1,354	1,524	1,826	1,499

	CON	BMI I	BMI II	BMI III
Mean	22,10	17,90	18,73	18,02
Std. Deviation	7,316	8,435	9,717	9,045
Std. Error of Mean	1,359	1,886	2,357	1,929

	CON	<15 years	>15 years
Mean	22,10	19,59	16,92
Std. Deviation	7,316	10,05	7,645
Std. Error of Mean	1,359	1,900	1,373

	R VALUE	P VALUE
WBC	0,0302879768514635	0,819863270480659
RBC	0,114301252677485	0,388682244718971
HGB	0,157405555452884	0,233800760304756
ALT	0,253782501854147	0,0524387098256345
AST	0,193441093029966	0,142114246314589
PLT	0,0249334401607663	0,851308804613478
CHOL	0,126320729429477	0,397503438512907
TG	0,214461568364573	0,147732384445719
HDL	0,157751516189022	0,31839629061402
LDL	-0,115476304384672	0,483906292872305
GLU	0,13600645907101	0,317563363224391
CRP	-0,0224047955736502	0,873479862916607
CRP	0,120805826702119	0,370711158359738
GFR	-0,00197929983506128	0,988341464251434
UREA	0,118081984738679	0,661201652077117
BUN	-0,228472416060177	0,408932337724004
UA	0,229960353307123	0,303230754740168

	R VALUE	P VALUE
PASI	0,172815737301901	0,202778793746647
BMI	-0,0344111477597437	0,795837174954566
AGE	0,140908411611202	0,287101157498191
PSORIASIS DURATION	0,0336798973983045	0,800085200646812

4 Kynurenine

4.1 Serum kynurenine

	CON	PSOR
Mean	22,10	18,18
Std. Deviation	7,316	8,894
Std. Error of Mean	1,359	1,158

	CON	PASI I	PASI II	PASI III
Minimum	1,047	2,087	0,8390	2,099
25% Percentile	1,503	2,148	1,904	2,168
Median	1,754	2,524	2,714	2,619
75% Percentile	2,073	3,450	3,585	3,012
Maximum	3,164	4,237	4,989	6,630
Range	2,117	2,150	4,150	4,531

	CON FEMALE	PSOR FEMALE	CON MALE	PSOR MALE
Minimum	1,047	0,8720	1,181	0,8390
25% Percentile	1,585	1,896	1,490	2,103
Median	1,995	2,380	1,735	2,636
75% Percentile	2,223	3,914	2,045	3,475
Maximum	3,164	4,920	3,089	6,630
Range	2,117	4,048	1,908	5,791

	CON	BMI I	BMI II	BMI III
Mean	1,047	0,8390	0,8720	1,730
Std. Deviation	3,164	4,915	6,630	4,989
Std. Error of Mean	2,117	4,076	5,758	3,259

	CON	<15 years	>15 years
Minimum	1,047	0,8390	0,8720
25% Percentile	1,503	1,931	2,127
Median	1,754	2,365	2,705
75% Percentile	2,073	3,163	3,586
Maximum	3,164	4,915	6,630
Range	2,117	4,076	5,758

	R VALUE	P VALUE
WBC	-0,00913691534422377	0,94572888458306
RBC	-0,220355675530861	0,0964832484744378
HGB	-0,0502600797327439	0,707903004940106
ALT	0,0124707540672359	0,925974641760675
AST	0,180255604782115	0,175729121966167
PLT	-0,156200968726066	0,241639369193732
CHOL	0,0500848116980091	0,740983835434201
TG	-0,0486661532385828	0,748076573231817
HDL	0,0368044928107825	0,819294044258704
LDL	-0,1987849743613	0,231523499681715
GLU	-0,0227071769770537	0,869294956163131
CRP	0,0257422784365222	0,85625167002922
CRP	-0,00834359309669087	0,951334904206254
GFR	-0,181513696432798	0,180622706467041
UREA	-0,141176470588235	0,601541074572301
BUN	-0,127470430593161	0,648786628898732
UA	-0,0867353767929442	0,701124890783381

	R VALUE	P VALUE
PASI	0,128481744735359	0,349869385436269
BMI	0,239260501128012	0,0704728349867343
AGE	0,482673775807917	1,24449145622689e-004
PSORIASIS DURATION	0,196674955241341	0,138947722019728

4.2 Urinary kynurenine/creatinine ratio concentration

	CON	PSOR
Minimum	1,130	0,9201
25% Percentile	2,221	2,990
Median	3,648	4,397
75% Percentile	6,472	7,362
Maximum	17,54	14,28
Range	16,41	13,36

	CON	PASI I	PASI II	PASI III
Minimum	1,130	2,409	1,597	0,9201
25% Percentile	2,221	3,081	3,374	2,142
Median	3,648	3,950	4,777	2,869
75% Percentile	6,472	6,061	7,665	7,431
Maximum	17,54	6,130	14,28	7,556
Range	16,41	3,721	12,68	6,636

	CON FEMALE	PSOR FEMALE	CON MALE	PSOR MALE
Minimum	1,300	2,585	1,130	0,9201
25% Percentile	2,483	3,470	2,018	2,734
Median	4,449	4,617	3,604	4,343
75% Percentile	6,472	7,982	6,528	7,362
Maximum	6,792	9,685	17,54	14,28
Range	5,491	7,100	16,41	13,36

	CON	BMI I	BMI II	BMI III
Minimum	1,130	0,9201	1,551	1,380
25% Percentile	2,221	2,142	2,890	3,382
Median	3,648	4,111	3,982	5,232
75% Percentile	6,472	7,287	6,200	7,548
Maximum	17,54	10,01	14,28	8,860
Range	16,41	9,089	12,73	7,480

	CON	<15 years	>15 years
Minimum	1,130	1,187	0,9201
25% Percentile	2,221	2,474	3,025
Median	3,648	4,134	5,350
75% Percentile	6,472	5,825	7,523
Maximum	17,54	9,685	14,28
Range	16,41	8,498	13,36

	R VALUE	P VALUE
WBC	0,0400939958823327	0,76714161302421
RBC	-0,150961137148432	0,262322039086939
HGB	-0,0208833293243902	0,877460389225231
ALT	-0,0787971566303254	0,560140591367218
AST	-0,0793625995714865	0,557320012328578
PLT	-0,105186387726837	0,436150694914378
CHOL	0,249059398675348	0,0950910233325565
TG	0,187571320657199	0,211930644870899
HDL	0,00915671644506909	0,954688964570065
LDL	0,181698783876641	0,27493428989274
GLU	0,367776720164968	0,00621969448313088**
CRP	0,00389166686418167	0,978377465301884
CRP	-0,349268263718702	0,00895879821173571
GFR	0,177925287281601	0,19373154133571
UREA	0,195604395604396	0,502204148341921
BUN	-0,00275864430716294	0,996422070727626
UA	0,009066977501416	0,969736798275628

	R VALUE	P VALUE
PASI	-0,0389981704288651	0,779494547183698
BMI	0,265690071497022	0,0457669542049144*
AGE	0,362731607496346	0,00555387287636633**
PSORIASIS DURATION	0,19832949842887	0,139162542882957

5 Kynurenic acid

5.1 Serum kynurenic acid

	CON	PSOR
Minimum	11,09	10,21
25% Percentile	26,32	38,72
Median	62,79	81,44
75% Percentile	77,46	129,3
Maximum	186,2	242,2
Range	175,1	232,0

	CON	PASI I	PASI II	PASI III
Minimum	11,09	16,71	10,21	23,66
25% Percentile	26,32	26,57	52,77	30,76
Median	62,79	77,03	88,87	38,43
75% Percentile	77,46	167,7	129,5	108,0
Maximum	186,2	242,2	206,1	147,3
Range	175,1	225,5	195,9	123,6

	CON FEMALE	PSOR FEMALE	CON MALE	PSOR MALE
Minimum	11,09	16,71	13,83	10,21
25% Percentile	29,42	36,17	26,07	38,95
Median	60,26	60,26	68,73	89,57
75% Percentile	68,65	124,7	86,54	130,0
Maximum	74,35	206,1	186,2	242,2
Range	63,26	189,4	172,4	232,0

	CON	BMI I	BMI II	BMI III
Minimum	11,09	32,54	10,21	16,71
25% Percentile	26,32	53,06	29,83	34,50
Median	62,79	85,29	79,76	60,26
75% Percentile	77,46	146,8	135,2	98,66
Maximum	186,2	185,3	242,2	206,1
Range	175,1	152,8	232,0	189,4

	CON	<15 years	>15 years
Minimum	11,09	10,21	23,66
25% Percentile	26,32	38,72	38,13
Median	62,79	88,87	67,31
75% Percentile	77,46	132,0	124,6
Maximum	186,2	206,1	242,2
Range	175,1	195,9	218,6

	R VALUE	P VALUE
WBC	0,137477533488506	0,299115884832715
RBC	0,146581711775275	0,26793869816827
HGB	0,18461181304017	0,161586949470374
ALT	0,035394105303156	0,790136204709875
AST	-0,0361146470354321	0,785964112358515
PLT	0,0991114763588762	0,455158486316736
CHOL	0,130511465208541	0,381902278597357
TG	0,112643479940735	0,45093678966869
HDL	0,131262006202235	0,407332636247279
LDL	0,0587252606104387	0,722504321414887
GLU	-0,0344385432507407	0,801059165535076
CRP	-0,0696703962568343	0,620094291996222
CRP	0,304414836287285	0,0213181528110446*
GFR	-0,196302117962608	0,143331744357514
UREA	0,152941176470588	0,571043583619626
BUN	0,0664282525626334	0,813943794154112
UA	-0,0215421197263521	0,299115884832715

	R VALUE	P VALUE
PASI	-0,184512316106634	0,173405395001683
BMI	-0,123818290753877	0,350139262853966
AGE	-0,0168104094515223	0,89943956373029
PSORIASIS DURATION	-0,0503641490277127	0,704823201859228

5.2 Urinary kynurenic acid/creatinine ratio concentration

	CON	PSOR
Minimum	0,01100	0,2400
25% Percentile	0,9900	2,300
Median	2,500	3,800
75% Percentile	3,950	4,900
Maximum	6,700	14,00
Range	6,689	13,76

	CON	PASI I	PASI II	PASI III
Minimum	0,01100	1,200	0,2400	0,4300
25% Percentile	1,095	3,000	2,300	1,200
Median	2,500	4,600	3,900	2,700
75% Percentile	3,925	5,900	5,600	4,500
Maximum	6,700	7,800	14,00	4,900
Range	6,689	6,600	13,76	4,470

	CON FEMALE	PSOR FEMALE	CON MALE	PSOR MALE
Minimum	0,07800	1,200	0,01100	0,2400
25% Percentile	0,7000	3,250	1,010	2,175
Median	2,300	4,600	2,650	3,350
75% Percentile	3,850	6,450	4,125	4,800
Maximum	6,700	14,00	6,500	9,900
Range	6,622	12,80	6,489	9,660

	CON	BMI I	BMI II	BMI III
Minimum	0,01100	0,4300	0,2400	1,200
25% Percentile	1,095	2,150	2,450	2,450
Median	2,500	3,600	3,750	4,200
75% Percentile	3,925	4,875	4,975	7,077
Maximum	6,700	9,900	6,100	14,00
Range	6,689	9,470	5,860	12,80

	CON	<15 years	>15 years
Minimum	0,01100	0,2400	0,4300
25% Percentile	1,095	2,175	2,300
Median	2,500	4,600	3,400
75% Percentile	3,925	5,825	4,500
Maximum	6,700	14,00	14,00
Range	6,689	13,76	13,57

	R VALUE	P VALUE
WBC	-0,0291176992590631	0,826713716112386
RBC	-0,221241138502155	0,0921886660104738
HGB	-0,0895411097599722	0,500044855172036
ALT	-0,103683895202656	0,434519855463699
AST	-0,216915415138552	0,0988973816677902
PLT	0,0843334753742919	0,525395391750385
CHOL	0,230600149339242	0,118891044923258
TG	0,0189443243217363	0,899423411250162
HDL	0,101335197201857	0,523112433448415
LDL	0,306270260890606	0,0579230584694568
GLU	-0,0168320226931859	0,902006487378598
CRP	-0,00584807134576528	0,966849676736074
CRP	-0,0591478953771336	0,662077614174494
GFR	-0,17273184914155	0,198831417942562
UREA	0,0309050856295262	0,91066328580683
BUN	-0,210244709120202	0,448244421638767
UA	0,0950090584418041	0,674066793629264

	R VALUE	P VALUE
PASI	0,0395415777649595	0,772320173615096
BMI	0,117197861832131	0,376693724941107
AGE	0,147867752339476	0,263716749687842
PSORIASIS DURATION	-0,0771393286654618	0,561437810834396

6. Quinolinic acid

6.1 Serum quinolinic acid

	CON	PSOR
Minimum	0,03500	0,08600
25% Percentile	0,6205	0,8480
Median	0,8075	1,360
75% Percentile	1,583	2,601
Maximum	3,698	9,897
Range	3,663	9,811

	CON	PASI I	PASI II	PASI III
Minimum	0,03500	0,5940	0,2150	0,08600
25% Percentile	0,6205	0,8480	0,7955	0,7910
Median	0,8075	2,141	1,360	1,161
75% Percentile	1,583	3,758	2,597	1,474
Maximum	3,698	5,339	9,897	6,801
Range	3,663	4,745	9,682	6,715

	CON FEMALE	PSOR FEMALE	CON MALE	PSOR MALE
Minimum	0,5150	0,2150	0,03500	0,08600
25% Percentile	0,6190	0,6340	0,6015	0,9090
Median	1,139	1,186	0,8050	1,390
75% Percentile	1,441	2,367	1,669	2,943
Maximum	3,698	8,227	3,214	9,897
Range	3,183	8,012	3,179	9,811

	CON	BMI I	BMI II	BMI III
Minimum	0,03500	0,08600	0,2150	0,3200
25% Percentile	0,6205	0,6463	0,8323	0,8195
Median	0,8075	1,303	1,242	1,400
75% Percentile	1,583	2,885	2,680	2,991
Maximum	3,698	4,222	9,897	8,227
Range	3,663	4,136	9,682	7,907

	CON	<15 years	>15 years
Minimum	0,03500	0,4610	0,08600
25% Percentile	0,6205	1,040	0,6150
Median	0,8075	1,419	1,162
75% Percentile	1,583	2,760	2,571
Maximum	3,698	9,897	8,227
Range	3,663	9,436	8,141

	R VALUE	P VALUE
WBC	0,070579983710617	0,595281039325633
RBC	0,178908605738077	0,175166735656483
HGB	0,248117808910181	0,058119141885103
ALT	-0,0541149543891228	0,68395676035664
AST	-0,0111797367646151	0,933025882697262
PLT	-0,0665517050041761	0,61650370563709
CHOL	0,0634342389604651	0,671857602152373
TG	0,154219795175534	0,300661541300152
HDL	0,045836238259742	0,773163312670481
LDL	0,0321976428864129	0,845719818299573
GLU	0,159231238704517	0,241117155424456
CRP	-0,156556798996116	0,262929818565445
CRP	0,0362522142063469	0,78891291047549
GFR	0,041775372305399	0,757668144083983
UREA	0,155882352941176	0,563485069300812
BUN	-0,0933586252231605	0,739749042000034
UA	-0,200681852187596	0,37052214557619

	R VALUE	P VALUE
PASI	-0,123122149428695	0,365980965612735
BMI	0,124899545594069	0,345914952247562
AGE	-0,0309019178961027	0,816274757542346
PSORIASIS DURATION	-0,218449378099876	0,0964762814408332

6.2 Urinary quinolinic acid/creatinine ratio concentration

	CON	PSOR
Minimum	0,1200	0,6400
25% Percentile	2,000	8,250
Median	2,600	16,50
75% Percentile	6,100	36,00
Maximum	240,0	150,0
Range	239,9	149,4

	CON	PASI I	PASI II	PASI III
Minimum	0,1200	1,900	0,6400	1,000
25% Percentile	2,000	4,200	8,625	8,300
Median	2,600	6,100	18,50	11,00
75% Percentile	6,100	24,00	39,75	29,00
Maximum	240,0	62,00	150,0	51,00
Range	239,9	60,10	149,4	50,00

	CON FEMALE	PSOR FEMALE	CON MALE	PSOR MALE
Minimum	1,300	0,6400	0,1200	0,9500
25% Percentile	2,100	10,45	1,625	7,950
Median	2,900	30,00	2,550	12,50
75% Percentile	6,050	61,50	18,25	27,50
Maximum	86,00	150,0	240,0	51,00
Range	84,70	149,4	239,9	50,05

	CON	BMI I	BMI II	BMI III
Minimum	0,1200	2,100	0,9500	0,6400
25% Percentile	2,000	8,400	5,525	6,550
Median	2,600	16,00	15,00	17,00
75% Percentile	6,100	47,00	30,75	33,50
Maximum	240,0	150,0	94,00	62,00
Range	239,9	147,9	93,05	61,36

	CON	<15 years	>15 years
Minimum	0,1200	0,6400	1,000
25% Percentile	2,000	4,125	9,025
Median	2,600	11,50	20,00
75% Percentile	6,100	22,25	43,50
Maximum	240,0	150,0	140,0
Range	239,9	149,4	139,0

	R VALUE	P VALUE
WBC	0,0358807876756009	0,789163127080934
RBC	-0,167141121026497	0,209826543334033
HGB	-0,240290637249645	0,0692349378368611
ALT	-0,0835245567238831	0,53305613412786
AST	-0,123280168739288	0,356549898530411
PLT	0,138392651866812	0,300193265437309
CHOL	-0,0981605752863733	0,511552611117675
TG	-0,184839197600239	0,213565076222016
HDL	0,049547553581781	0,755340125409313
LDL	-0,146576175792528	0,373246491216243
GLU	0,0597241318635319	0,664913410827971
CRP	-0,00375755250892327	0,97890838957466
CRP	-0,373999603575138	0,0045188534450847**
GFR	0,235826003599837	0,0801651368966824
UREA	-0,241176470588235	0,36692610060808
BUN	-0,285461950201587	0,300080805401242
UA	0,122768058205782	0,586244142496314

	R VALUE	P VALUE
PASI	0,0791662913471242	0,565608832157141
BMI	-0,0216772573145887	0,871688419485958
AGE	0,0359242733540313	0,788913485336644
PSORIASIS DURATION	0,241092097101071	0,0682839055895241
